# Age quadratically affects intestinal calcium and phosphorus transporter gene expression in broiler chickens

**DOI:** 10.5713/ab.22.0058

**Published:** 2022-05-02

**Authors:** Xianliang Lv, Junfang Hao, Lihua Wu, Mengyuan Liu, Lei He, Yingying Qiao, Yanyan Cui, Guan Wang, Chunmei Zhang, Hongxia Qu, Jincheng Han

**Affiliations:** 1 College of Animal Science and Technology, Henan Agricultural University, Zhengzhou 450046, China; 2 Department of Animal Science, College of Biology and Food, Shangqiu Normal University, Shangqiu 476000, China; 3 College of Life Sciences, Henan Normal University, Xinxiang 453007, China; 4 Department of Biochemistry and Biotechnology, Sumy National Agrarian University, Sumy 40000, Ukraine

**Keywords:** Age, Broiler Chicken, CaBP-D28k, NaPi-IIb, PMCA1b, PiT-1

## Abstract

**Objective:**

This research aimed to evaluate the effects of age on growth, tibia development, and intestinal calcium (Ca) and phosphorus (P) transporter gene expressions in broiler chickens.

**Methods:**

A total of 224 male Arbor Acres broilers were fed with nutrient-adequate diets and reared in eight cages (28 broilers per cage). Eight broilers (one broiler per cage) were selected and killed at 5, 10, 15, 20, 25, 30, 35, and 40 days of age, respectively.

**Results:**

Body weight continuously increased with age of broiler chickens from 5 to 40 days. The bone weight, ash weight, diameter, and length of the tibia also increased with broiler age. By contrast, the tibia ash, Ca, and P percentages quadratically changed with age (p<0.001), and the highest values of mineral contents were observed at 20, 25, and 25 days of age, respectively. The mRNA abundances of calcium-binding protein 28-kDa (CaBP-D28k), sodium-calcium exchanger 1 (NCX1), and plasma membrane ATPase 1b (PMCA1b) increased from 5 to 25 days and then decreased up to 40 days. Similar results were noted in the mRNA abundances of IIb sodium-phosphate cotransporter (NaPi-IIb), inorganic phosphate transporter 1 (PiT-1), inorganic phosphate transporter 2 (PiT-2), nuclear vitamin D receptor (nVDR), and membrane vitamin D receptor (mVDR). The mRNA abundances of Ca and P transporters and VDRs were the highest at 25 days of age.

**Conclusion:**

These data indicate that age quadratically affects intestinal Ca and P transporter gene expression and mineral absorption capacity in broiler chickens.

## INTRODUCTION

Calcium (Ca) and phosphorus (P) are the most important and abundant mineral components of bones and teeth of animals. Feed is the primary source of Ca and P in animals. These components are mainly absorbed in the intestines, re-absorbed in the kidney, and retained in the bone.

In recent years, several Ca transporters have been observed in broiler chicken intestines, which include calcium-binding protein 28-kDa (CaBP-D28k), sodium-calcium exchanger 1 (NCX1), and plasma membrane ATPase 1b (PMCA1b) [1,2]. CaBP-D28k exists in poultry enterocyte cytoplasm, whereas NCX1 and PMCAlb lie in the basal membrane [3]. Their main responsibilities are to transport Ca from the intestinal cell into the blood. The mRNA abundances of duodenal CaBP-D28k and PMCAlb of laying hens are higher than those in other intestinal segments [4].

Three P transporters have been detected in the apical membrane of rat enterocytes, including IIb sodium-phosphate cotransporter (NaPi-IIb), inorganic phosphate transporter 1 (PiT-1), and 2 (PiT-2) [5,6]. Their duty is to transport P from the intestinal cavity into the enterocyte. The three P transporter genes have been cloned in broiler chicken intestines [7].

Intestinal Ca and P absorption is regulated by dietary vitamin D after binding to vitamin D receptor (VDR). Two VDRs (i.e., nuclear VDR [nVDR] and membrane VDR [mVDR]) have been reported in animal intestines. The nVDR lies in the nucleus of enterocytes, but the mVDR exists in the basal membrane. The mVDR is also named membrane-associated rapid response steroid-binding receptor [8].

Research in mammals has shown that intestinal Ca and P absorption changes with age. The capacity of intestinal Ca absorption and the ability of vitamin D to stimulate calbindin-D9k (CaBP-D9k) and *PMCA1b* gene expression decrease with age in rats [9–11]. Similarly, P absorption by the intestinal apical membrane and *NaPi-IIb* gene expression slow down with age in rats [12]. Furthermore, the expression level of VDR and its capacity to bind vitamin D in the intestines decrease with age in rats [13]. These data indicated that age affects the capacity of Ca and P absorption in the mammalian intestines.

Modern broiler chickens are fed with a high-nutrient-concentration diet and grow rapidly. However, broiler leg bone development is relatively slow. The incidence of tibial chondrodysplasia is high in broiler production. Therefore, this study was conducted to explore the regularity of chicken skeletal development and intestinal Ca and P absorption and to provide a theoretical basis for improving the bone development of broilers.

## MATERIALS AND METHODS

### Animals and diet

The procedures used in this animal experiment were carried out according to the guidelines of the Animal Care and Use Committee of Henan Agricultural University and Shangqiu Normal University.

A total of 224 male Arbor Acres broilers were fed with nutrient-adequate pellet diets [14] (Table 1) and reared in eight stainless-steel cages (width 280 cm, depth 70 cm, and height 35 cm) with 28 broilers per cage. Twenty hours of lighting was provided on days 1 to 40. The temperature of the feeding room was 32°C for 1 to 3 days, 30°C for 4 to 7 days, 28°C for 8 to 20 days, and 26°C for 21 to 40 days. The room humidity was controlled at 50% to 60%.

### Sample collection

Eight broilers (one broiler per replicate cage) were randomly selected, weighed, and killed at 5, 10, 15, 20, 25, 30, 35, and 40 days of age, respectively. The tibia was removed, collected, and kept after broilers were killed. The bone weight of the tibia was measured after it was dried at 105°C for 24 h. The ash weight of the tibia was determined when the bone was burned in a muffle furnace (Selecta, Barcelona, Spain) at 650°C for 48 h. Ca contents in the diet and tibia were analyzed by the ethylenediamine tetraacetic acid (EDTA) titration method. Total P contents were measured using the photometric method after reaction with ammonium molybdate and ammonium metavanadate. The ash, Ca, and P percentages were the ratios of their weight to the bone weight. The duodenum was isolated after the broilers were euthanized. Duodenal mucosa was scraped with a glass slide, collected in a centrifuge tube, and stored in liquid nitrogen [2].

### RNA extraction and real-time polymerase chain reaction

Total RNA extraction from the duodenal mucosa samples was implemented by RNAiso Plus Kit under the manufacturer’s recommendation (Takara Biotechnology Co. Ltd., Dalian, Liaoning, China). RNA concentration was analyzed with a spectrophotometer. The cDNA was reverse-transcribed from RNA using PrimeScript RT reagent Kit under the manufacturer’s instructions (Takara Biotechnology Co. Ltd., China). Real-time polymerase chain reaction (PCR) analysis was carried out to determine the mRNA abundances of Ca and P transporters (i.e., CaBP-D28k, PMCA1b, NCX1, NaPi-IIb, PiT-1, and PiT-2) and VDRs (i.e., nVDR and mVDR). Glyceraldehyde 3-phosphate dehydrogenase (GAPDH) was used as a reference gene. PCR primers (Table 2) were synthesized by Shanghai Sangon Biotech Co., Ltd. Gene expression was determined using the TB Green Premix Ex Taq II Kit (Takara Biotechnology Co. Ltd., China) and Roche Lightcycler 480 Real-time PCR system (Roche Ltd., Basel, Switzerland). The PCR product specificities were verified by melting curve analysis, which was implemented under the following conditions: 95°C for 60 s, 40 cycles of 95°C for 10 s, 60°C for 30 s, and 72°C for 30 s. The relative mRNA abundance of target genes relative to that of the *GAPDH* gene was calculated by the 2^−ΔΔCt^ method [15]. The average ΔCt value from broilers at 5 days of age was used as the calibrator of each gene.

### Statistical analysis

Eight replicate cages were used as experimental units. Data was analyzed by one-way analysis of variance of SAS software [16]. Polynomial comparisons was used to evaluate the effects of age on tibia mineral contents and gene expression of Ca and P transporters and VDRs. The level of significance was set at p<0.05.

## RESULTS

### Growth performance

**Figure 1:**
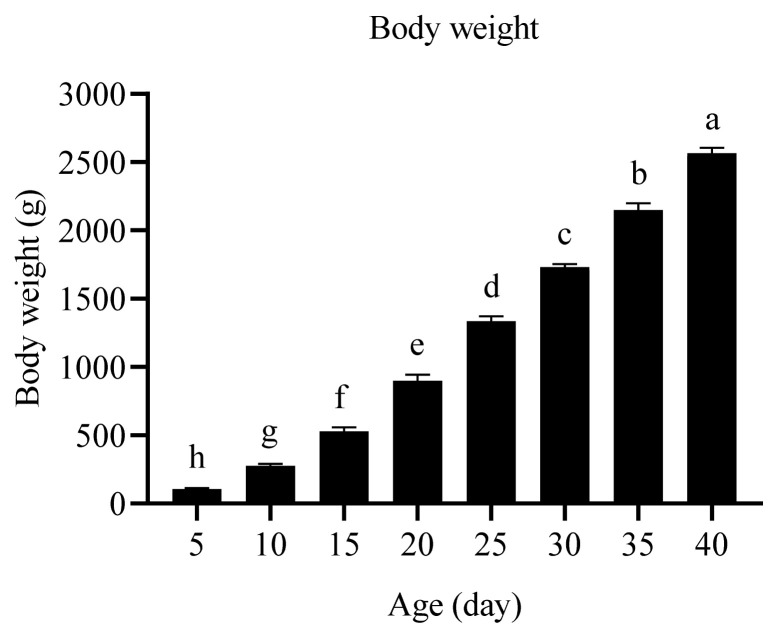
Effects of age on the body weight of broilers from 5 to 40 days. The values are the means of eight broilers (n = 8) and presented as mean±standard deviation. ^a–h^ Values with different superscripts are significantly different (p<0.05).

The body weights of Arbor Acres broilers at 5, 10, 15, 20, 25, 30, 35, and 40 days of age were 105, 275, 528, 898, 1,336, 1,732, 2,148, and 2,566 g/bird, respectively. With the increase of broiler age, the body weight was enhanced (Figure 1).

### Bone mineralization

**Figure 2:**
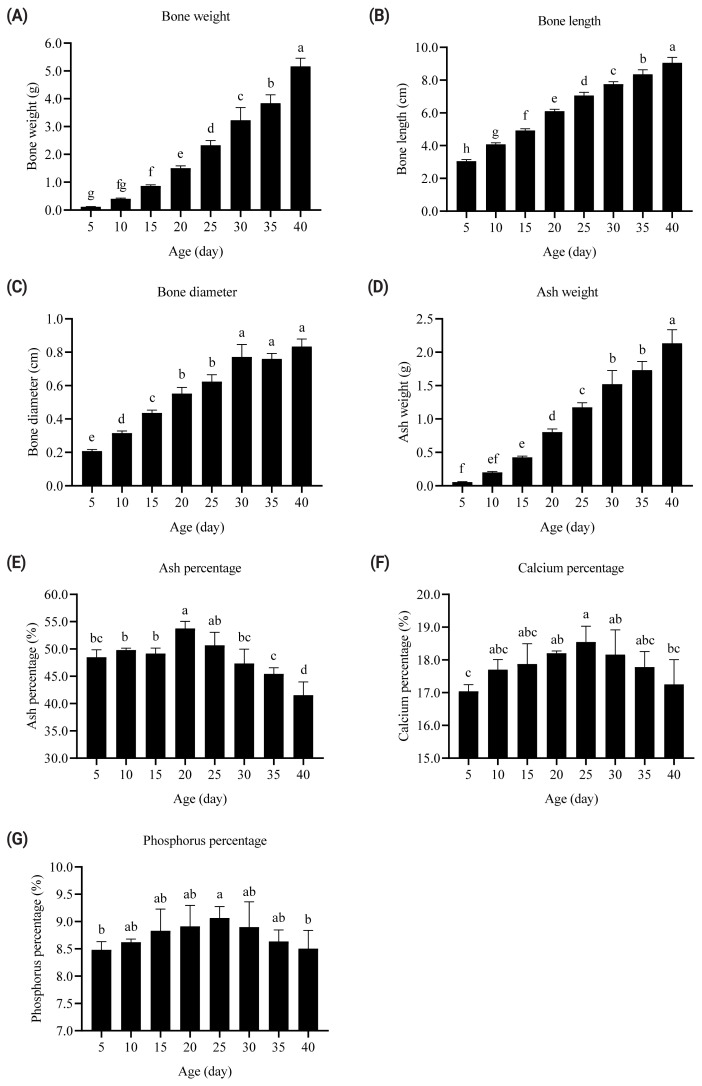
Effects of age on the bone weight (A), length (B), diameter (C), ash weight (D), ash percentage (E, quadratic p<0.001), calcium percentage (F, quadratic p<0.001), and phosphorus percentage (G, quadratic p<0.001) in the tibia of broilers from 5 to 40 days. The values are the means of eight broilers (n = 8) and presented as mean±standard deviation. ^a–h^ Values with different superscripts are significantly different (p<0.05).

The bone weight, ash weight, diameter, and length of the tibia were continuously enhanced with increasing age of broilers from 5 to 40 days (Figure 2A, 2B, 2C, and 2D). By contrast, the ash, Ca, and P percentage contents of the tibia quadratically changed with age (p<0.001; Figure 2E, 2F, and 2G). The ash, Ca, and P percentage contents increased from 5 to 20 days and decreased from 25 to 40 days. The tibia ash percentage of broilers at 5, 10, 15, 30, 35, and 40 days of age was lower than that of broilers at 20 days of age (p<0.05). The tibia Ca and P percentages of 5- and 40-day-old broilers were lower than that of 25-day-old broilers (p<0.05). The tibia ash, Ca, and P percentage contents were the highest in broilers at 20, 25, and 25 days of age, respectively.

### Gene expression

**Figure 3:**
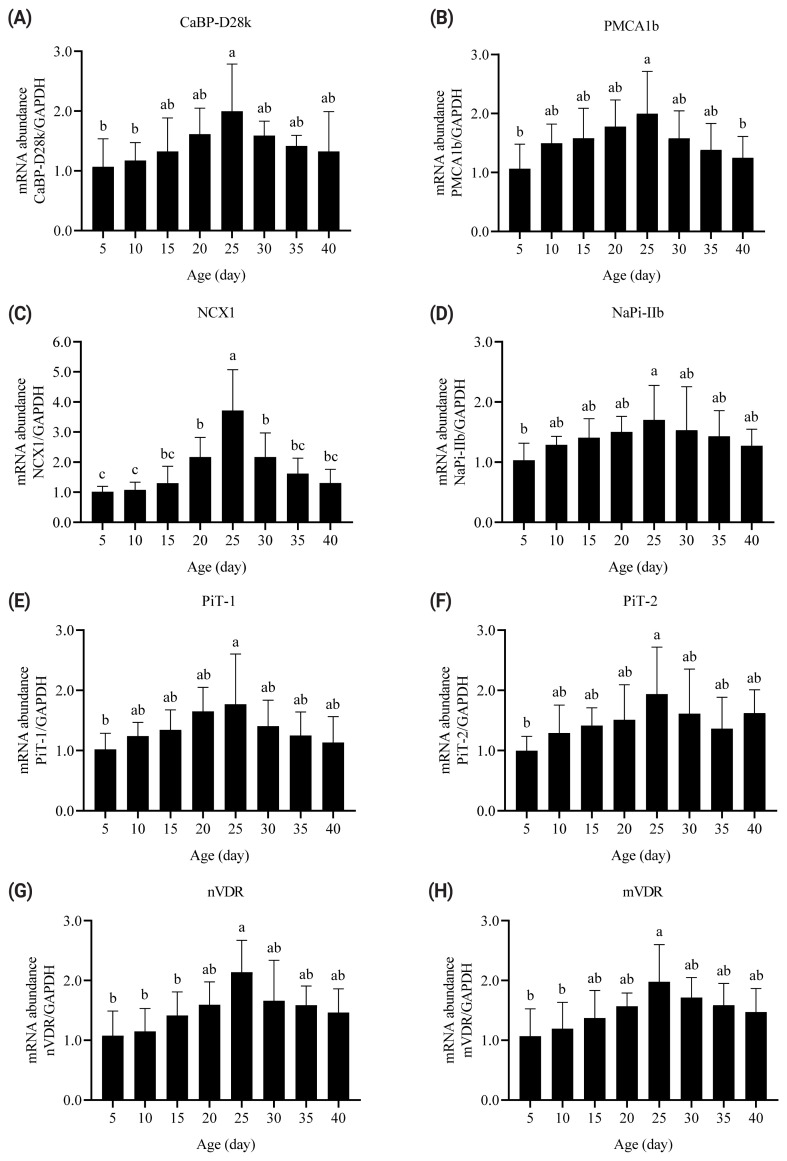
Effects of age on the mRNA abundances of duodenal CaBP-D28k (A, quadratic p = 0.002), PMCA1b (B, quadratic p<0.001), NCX1 (C, quadratic p<0.001), NaPi-IIb (D, quadratic p = 0.003), PiT-1 (E, quadratic p<0.001), PiT-2 (F, quadratic p = 0.034), nVDR (G, quadratic p<0.001), and mVDR (H, quadratic p = 0.002) in broilers from 5 to 40 days. The values are the means of eight broilers (n = 8) and presented as mean±standard deviation. ^a–c^ Values with different superscripts are significantly different (p<0.05). CaBP-D28k, calcium-binding protein 28-kDa; PMCA1b, plasma membrane calcium ATPase 1b; NCX1, sodium-calcium exchanger 1; NaPi-IIb, IIb sodium-phosphate cotransporter; PiT-1, inorganic phosphate transporter 1; PiT-2, inorganic phosphate transporter 2; nVDR, nuclear vitamin D receptor; and mVDR, membrane vitamin D receptor.

CaBP-D28k, PMCA1b, and NCX1 are Ca transporters in enterocytes. Their mRNA abundances quadratically changed with broiler age (p = 0.002, p<0.001, and p<0.001, Figure 3A, 3B, and 3C). The increase in age from 5 to 25 days increased the mRNA abundances of the three Ca transporters. By contrast, further increasing the age from 25 to 40 days decreased their mRNA abundances. The mRNA abundance of duodenal CaBP-D28k in 5- and 10-day-old broilers was lower than that in 25-day-old broilers (p<0.05). The mRNA abundance of PMCA1b in 25-day-old broilers was higher than those in 5- and 40-day-old broilers (p<0.05). The mRNA abundance of NCX1 was the highest in 25-day-old broilers.

NaPi-IIb, PiT-1, and PiT-2 are intestinal P transporters. Their mRNA abundances in the duodenum quadratically changed with age (p = 0.003, p<0.001, and p = 0.034; Figure 3D, 3E, and 3F). Increasing broiler age from 5 to 25 days increased the mRNA abundances of NaPi-IIb, PiT-1, and PiT-2. The mRNA abundances of the three P transporters in 5-day-old broilers were lower than those in 25 day-old-broilers (p<0.05). Further increasing the age from 25 to 40 days decreased the mRNA abundances of the three P transporters.

Two VDRs (i.e., nVDR and mVDR) in the duodenum were measured. A quadratic relationship existed between broiler age and the mRNA abundances of the two VDRs (p< 0.001 and p = 0.002; Figure 3G and 3H). The mRNA abundances of nVDR and mVDR increased from 5 to 25 days and then decreased. The mRNA abundances of the two VDRs in 5- and 10-day-old broilers were lower than those in 25-day-old broilers (p<0.05).

## DISCUSSION

Dietary nutrients are mainly retained in the meat, fat, and bone of chickens. The weight of meat, fat, bone, blood, gastrointestinal tract, and feather is enhanced with the increase of broiler age from 1 to 70 days [17]. Broilers at 1 to 40 days of age were fed with complete compound feed in this research [14]. The body weight increased when dietary nutrients were absorbed in the small intestine and retained in the meat and bone.

The increases in tibia weight and ash weight come from the enhancement of feed intake and retained minerals with age [18,19]. The starter phase (days 1 to 20) had a faster growth rate in the leg bones than the grower-finisher phase (days 21 to 40) in this research. The bone weight of 20-day-old broilers was 12.5-fold higher than that of 5-day-old broilers. In comparison, the bone weight of 40-day-old broilers was 2.2-fold higher than that of 25-day-old broilers. Similar results were noted in the tibia ash weight, diameter, and length. By contrast, tibia ash, Ca, and P contents quadratically changed with broiler age. The mineral contents increased from 5 to 20 days and decreased from 25 to 40 days. The tibia ash, Ca, and P percentages were the highest in broilers at 20, 25, and 25 days of age, respectively. Dietary Ca and NPP levels were 10.0 and 4.5 g/kg in starters (days 1 to 20) and 9.0 and 3.5 g/kg in grower-finishers (days 21 to 40), respectively [20]. These data suggest that age affects the mineral requirement of broilers and the capacity of mineral absorption in the intestines and retention in the bones. The relationship between age and intestinal mineral absorption should be clarified. Thus, the duodenal Ca and P transporter gene expression was evaluated in broilers at different ages.

CaBP-D28k and CaBP-D9k are Ca transporters and expressed in the intestines of poultry and mammals, respectively. Broiler age influences the mRNA and protein expressions of intestinal CaBP-D28k [1,21]. Increasing the age of laying hens from 12 to 23 weeks increased the mRNA abundance of intestinal CaBP-D28k [22]. By contrast, the mRNA and protein abundances of duodenal CaBP-D9k in young rats (2 months) were higher than those in adult rats (12 months) [23]. This study showed that the mRNA abundance of CaBP-D28k quadratically changed with broiler age. The mRNA abundance of CaBP-D28k increased from 5 to 25 days and reduced from 25 to 40 days. These data indicated that the gene expression of CaBP-D28k and the ability of Ca transport in intestinal cells were enhanced from hatching to grower phase and declined from the grower to finisher phase of broilers.

Studies in mammals have shown that the gene expression of PMCA1b changes with animal age. The mRNA abundance of duodenal PMCA1b in young rats (2 months) are 3 to 4 times higher than those in adult (12 months) and old (27 months) rats [24]. Similarly, the protein abundance of PMCA1b in both the duodenum and ileum of rats decreases with the increase of age at 2, 12, and 24 months [11]. The mRNA and protein abundances of intestinal PMCA1b also change with age of laying hens [25]. This research showed that the mRNA abundance of duodenal PMCA1b increased from 5 to 25 days of age and decreased upto 40 days of age in broiler chickens. The mRNA abundance was the highest in broilers at 25 days of age. These data revealed that the ability of PMCA1b in the basolateral membrane to transport Ca in the intestinal cells to the blood changes with animals’ age.

NCX1 is a sodium and Ca exchanger. Dietary Ca deficiency upregulates the gene expression of NCX1 in chick intestines [2,26]. The relationship between broiler age and intestinal *NCX1* gene expression has not been reported. The present research showed that the mRNA abundance of duodenal NCX1 quadratically enhanced with age, and the highest value was detected in broilers at 25 days of age. These data suggest that the capacity of Na and Ca ion exchanges between the intestinal cell and the blood in poultry changes with age.

NaPi-IIb is the main P transporter in animal intestines. The mRNA levels of intestinal NaPi-IIb decrease in rats at 2 to 15 weeks of age [12,27]. Studies in poultry have shown that broiler age affects the mRNA abundance of NaPi-IIb in the duodenum and jejunum [21]. The present research showed that the mRNA abundance of NaPi-IIb quadratically changed with broiler age. Increasing age from 5 to 25 days increased the mRNA abundance of NaPi-IIb, but further increasing age from 25 to 40 days decreased its abundance. These data revealed that age affects P transporter gene transcription and the capacity of P absorption in the apical membrane of enterocytes.

PiT-1 and PiT-2 exist in the apical membrane of rat intestinal cells [5,6]. The mRNA abundances of PiT-1 and PiT-2 are lower than that of NaPi-IIb in rat intestines [27]. Gene expressions of intestinal PiT-1 and PiT-2 are not sensitive to rat ages (weeks 4, 9, and 15) [27]. By contrast, the present research showed that the mRNA abundances of duodenal PiT-1 and PiT-2 quadratically increased with age, and the highest values were noted in broilers at 25 days of age. These data indicated that broiler age influences intestinal *PiT-1* and *PiT-2* gene expression. The different response of these two P transporter gene expression to age between rats and broilers can be attributed to the difference of species and sampling time points.

Vitamin D regulates intestinal mineral absorption after binding VDRs. *VDR* gene expression and its capacity to bind 1,25-(OH)_2_-D_3_ decrease in the intestines of aged rats (24 months) compared with young rats (3 months) [13], in which *VDR* gene expression in rats from birth to youth is not examined. A quadratic relationship was detected in broiler age and the mRNA abundances of nVDR and mVDR in this research. The mRNA abundances of the two VDRs were increased from 5 to 25 days and then decreased. These data indicated that age influences gene transcription of intestinal VDRs, and the highest expression level of VDRs existed in the grower phase of broilers.

## CONCLUSION

Body weight, as well as bone weight, ash weight, diameter, and length of the tibia increased with age in broiler chickens from 5 to 40 days. The mRNA abundances of intestinal CaBP-D28k, PMCA1b, NCX1, NaPi-IIb, PiT-1, PiT-2, nVDR, and mVDR increased from 5 to 25 days. Thus, intestinal Ca and P absorption capacity and tibia Ca and P contents elevated. By contrast, the mRNA abundances of Ca and P transporters reduced from 25 to 40 days. Intestinal mineral transport capacity and leg bone mineral contents decreased accordingly. These data indicate that the gene expression of Ca and P transporters and the ability of mineral absorption in the intestines are quadratically changed with age of broiler chickens.

## Figures and Tables

**Table 1:** Experimental diet composition (as-fed basis)

Item	Days 1 to 20	Days 21 to 40
Ingredient (g/kg)		
Corn	562.9	633.1
Soybean meal (43% crude protein)	331.8	274.9
Soybean oil	27.9	29.8
Soy protein powder (65% crude protein)	36.7	27.5
Limestone	13.1	14.6
Dicalcium phosphate	19.5	13.4
L-Lysine·HCl (98%)	1.3	1.5
DL-Methionine (98%)	1.5	0.9
Trace mineral premix^1)^	0.1	0.1
Vitamin premix^2)^	0.2	0.2
Choline chloride (50%)	2.0	1.0
Sodium chloride	3.0	3.0
Nutrient composition (g/kg)		
Metabolizable energy (kcal/kg)	2,975	3,053
Crude protein	215.0	190.7
Calcium (Ca)	10.0	9.0
Analyzed Ca	9.8	8.7
Total phosphorus (tP)	6.9	5.7
Analyzed tP	6.8	5.9
Non-phytate phosphorus (NPP)	4.5	3.5
Lysine	11.1	10.0
Methionine	5.1	4.2
Tryptophan	2.8	2.4
Threonine	8.7	7.6

1) The trace mineral premix provided the following (per kilogram of diet): 100 mg iron, 8 mg copper, 80 mg manganese, 100 mg zinc, 0.15 mg selenium, and 0.35 mg iodine.

2) The vitamin premix provided the following (per kilogram of diet): 7,000 IU vitamin A, 25 μg cholecalciferol, 19 IU vitamin E, 0.6 mg menadione, 2.2 mg thiamine, 8.5 mg riboflavin, 11.0 mg pantothenic acid, 36 mg niacin, 3.8 mg pyridoxine, 0.20 mg biotin, 0.56 mg folic acid, and 0.01 mg vitamin B_12_.

**Table 2:** Primer sequences used in real-time quantitative polymerase chain reaction

Gene	Accession number	Forward	Reverse
*CaBP-D28k*	NM_205513.1	5′-AGATCTGGCACCACTACGAC-3′	5′-TGAGCAAGCTCAACGATTCCT-3′
*PMCAlb*	NM_001168002.3	5′-AGCTCAAGATGGTGCAGCTA-3′	5′-AACAAACCTGCTTTGCCAATCT-3′
*NCX1*	NM_001079473.1	5′-TCACCTTCTTCTTCTTCCCAATCT-3′	5′-GCAACCTTTCCGTCCATCTC-3′
*NaPi-IIb*	NM_204474.1	5′-TCGGTCCGTTCACTCTGTTG-3′	5′-GCCACGTTGCCTTTGTGATT-3′
*PiT-1*	XM_015297502.1	5′-GGCTCCGTGCTTCTGG-3′	5′-CATTTGACGCCTTTCTGC-3′
*PiT-2*	NM_001305398.1	5′-GCAGCAGATACATCAACTC-3′	5′-ATTTCCACTCCACCCTC-3′
*nVDR*	AF011356.1	5′-AAGTCATCGACACCCTCCTG-3′	5′-GCCAAAGACATCGTTGGAGT-3′
*mVDR*	NM_204110.3	5′-GCTGTTGCTAGCCGGAAAAC-3′	5′-TCCAGAGCCTTTCCATCACG-3′
*GAPDH*	NM_204305.1	5′-GAACATCATCCCAGCGTCCA-3′	5′-ACGGCAGGTCAGGTCAACAA-3′

*CaBP-D28k*, calcium-binding protein 28-kDa; *PMCA1b*, plasma membrane calcium ATPase 1b; *NCX1*, sodium-calcium exchanger 1; *NaPi-IIb*, IIb sodium-phosphate cotransporter; *PiT-1*, inorganic phosphate transporter 1; *PiT-2*, inorganic phosphate transporter 2; *nVDR*, nuclear vitamin D receptor; *mVDR*, membrane vitamin D receptor; *GAPDH*, glyceraldehyde-3-phosphate dehydrogenase.
